# From bench to bedside, boswellic acids in anti-inflammatory therapy — mechanistic insights, bioavailability challenges, and optimization approaches

**DOI:** 10.3389/fphar.2025.1692443

**Published:** 2025-11-18

**Authors:** Chuhang Peng, Yujia Yang, Yuzhi Wang, Bao Gong, Xiuting Sun, Xinquan Yang

**Affiliations:** 1 Institute of Medicinal Plant Development, Chinese Academy of Medical Sciences & Peking Union Medical College, Beijing, China; 2 Hainan Provincial Key Laboratory of Resources Conservation and Development of Southern Medicine, Hainan Branch of the Institute of Medicinal Plant Development, Chinese Academy of Medical Sciences & Peking Union Medical College, Haikou, China

**Keywords:** boswellic acids, anti-inflammatory mechanisms, bioavailability, pharmacokinetics, clinical trials

## Abstract

Boswellic acids (BAs), a group of pentacyclic triterpenoids derived from the gum resin of Boswellia species, exhibit promising anti-inflammatory potential through diverse mechanisms. This review provides a comprehensive and structured summary of BAs’ anti-inflammatory actions, spanning key signaling pathways including NF-κB, MAPK, 5-LOX, COX-2, and NLRP3 inflammasome, as well as their modulation of cytokines, immune cell activity, and oxidative stress. We further highlight recent progress in molecular docking and dynamic simulations that elucidate BA–protein interactions at the structural level. The review integrates evidence from preclinical and clinical studies, with detailed pharmacological parameters such as model types, dose ranges, and control settings. Challenges related to BAs’ poor solubility and limited bioavailability are critically addressed. Recent advances in delivery systems, including nanoparticles, micelles, phytosomes, and ligand-targeted carriers—are summarized with mechanistic insight. Safety, toxicity, and formulation limitations are also discussed to provide a balanced perspective on their clinical translation. Overall, this review aims to clarify how BAs exert multi-target immunomodulatory effects and proposes directions for future research and therapeutic development.

## 1 Introduction

### 1.1 Inflammation and its pathophysiological significance

Inflammation is a complex biological response of body tissues to infection, injury, or other harmful stimuli, aimed at eliminating pathogens and damaged cells, and promoting tissue repair. It encompasses a broad spectrum of responses that vary depending on the triggering stimulus and subsequent signaling events, involving a diverse array of pathogenic cells, stromal cells, and components of both the innate and adaptive immune systems (Paus et al., 2018). However, if uncontrolled or persistent, inflammation can contribute to the development of autoimmune or autoinflammatory diseases, neurodegenerative disorders, and even cancer (Mantovani et al., 2008; Dinarello, 2010). Inflammation is generally classified into two types:

Acute inflammation: This is typically induced by infections, tissue injury, or exposure to toxins, and is characterized by local vasodilation, leukocyte infiltration, and the release of pro-inflammatory cytokines derived from macrophages, such as TNF-α, IL-1β, and IL-6 (Paus et al., 2018).

Chronic inflammation: This form persists over time and is often associated with autoimmune diseases, metabolic disorders, and cancer. It is marked by continuous infiltration of immune cells—primarily macrophages and T lymphocytes—and sustained tissue damage (Medzhitov, 2008).

### 1.2 Mechanisms of inflammatory response

Inflammation is a highly dynamic and multifaceted process that involves both innate and adaptive immune mechanisms. It is initiated when host pattern recognition receptors (PRRs) detect pathogen-associated molecular patterns (PAMPs) or damage-associated molecular patterns (DAMPs), leading to activation of transcription factors such as NF-κB and induction of pro-inflammatory mediators (Newton and Dixit, 2012; Zindel and Kubes, 2020).

Innate immunity: Toll-like receptors (TLRs) play a central role by sensing microbial ligands and triggering downstream NF-κB and MAPK pathways, thereby promoting cytokine release (O'Neill et al., 2013). In addition, activation of the NLRP3 inflammasome results in caspase-1–mediated maturation of IL-1β and IL-18, which amplify local and systemic inflammation (Guo et al., 2015).

Adaptive immune cells also play critical roles in sustaining and shaping inflammation. Distinct CD4^+^ T helper (Th) cell subsets mediate different inflammatory programs: Th1 responses are typically associated with IFN-γ–driven cellular immunity, Th17 responses with IL-17–mediated recruitment of neutrophils and chronic tissue inflammation, while regulatory T cells (Tregs) limit excessive immune activation through suppressive cytokines such as IL-10 and TGF-β (Paul and Zhu, 2010). B cells further contribute to chronic inflammation by producing antibodies and immune complexes that perpetuate tissue injury and amplify inflammatory cascades (Coussens and Werb, 2002).

Cytokine and chemokine networks. Key mediators such as IL-1, IL-6, TNF-α, and IFN-γ are pivotal in driving both acute and chronic inflammatory responses (Dinarello, 2010). Chemokines further guide neutrophils, monocytes, and lymphocytes to inflamed tissues, ensuring amplification and persistence of the immune response (Mantovani et al., 2008).

Oxidative and stress pathways. Reactive oxygen species (ROS) generated by NADPH oxidases and mitochondria function as signaling messengers that activate MAPKs (ERK, JNK, p38) and JAK-STAT cascades, further regulating inflammatory gene expression (Morgan and Liu, 2011). Dysregulation of these signaling networks is closely associated with the transition from acute to chronic inflammation and contributes to disease pathogenesis (Nathan and Ding, 2010).

Inflammation plays a pivotal role in the pathogenesis of numerous diseases, such as rheumatoid arthritis, inflammatory bowel disease, cardiovascular disorders, and neurodegenerative diseases. Consequently, anti-inflammatory therapies have become a central focus of medical research. The most used clinical anti-inflammatory agents include nonsteroidal anti-inflammatory drugs (NSAIDs), glucocorticoids (GCs), biological agents, and Janus kinase (JAK) inhibitors. These drugs effectively control inflammatory responses (Staa et al., 2002; Beaulieu and Morand, 2011; Schjerning et al., 2020); however, their long-term use is often limited by a range of adverse effects, development of drug resistance, and safety concerns. These limitations have restricted their widespread clinical use. For instance, the inhibition of COX-1 by NSAIDs can damage gastric mucosa, increasing the risk of gastric ulcers and bleeding. Additionally, some COX-2 inhibitors, such as celecoxib, have been associated with an elevated risk of cardiovascular events (Bindu et al., 2020). Although glucocorticoids are potent anti-inflammatory agents, prolonged administration can cause osteoporosis, hypertension, diabetes, and immunosuppression (Staa et al., 2002). Biologics, while targeting specific inflammatory mediators and thereby reducing broad immunosuppression, are costly and may induce drug resistance or immune dysregulation in some patients after long-term use (Dinarello, 2010). Moreover, JAK inhibitors, a relatively recent class of anti-inflammatory drugs, may increase the risk of infections due to their extensive effects on the immune system.

In recent years, natural products have emerged as a significant focus in anti-inflammatory research due to their broad-spectrum multi-target effects and relatively low side effects. Among these, boswellic acids (BAs) are a class of pentacyclic triterpenoids extracted from plants of the Boswellia genus (Efferth and Oesch, 2022).

### 1.3 Overview of boswellic acids

Frankincense refers to the oleo-gum resins obtained from trees of the genus *Boswellia* Roxb. ex Colebr. (family Burseraceae), (Howes, 1949). The genus *Boswellia* Roxb. ex Colebr. (family Burseraceae) comprises 23 Accepted species distributed across India, the Arabian Peninsula, and Africa (Plants of the World Online, 2025).

Among them, *Boswellia serrata* Roxb. ex Colebr., *Boswellia. frereana* Birdw., *Boswellia. sacra* Flueck., and *Boswellia. papyrifera* (Caill.) Hochst. are the principal sources of medicinal frankincense. All species names have been validated following *Plants of the World Online* (Boswellia serrata Roxb, 2025; Boswellia sacra Flück, 2025; Boswellia papyrifera (Caill.) Hochst (2025); Boswellia frereana Birdw, 2025).

However, only a small subset of these metabolites exhibits significant medicinal value. Indian frankincense is the gum resin collected from the *Boswellia serrata* Roxb. ex Colebr. (Burseraceae), while African frankincense contains gum resins from *Boswellia carteri* Birdw. (Burseraceae) and *Boswellia*. *frereana* Birdw. (Burseraceae) (Büchele et al., 2003). The use of frankincense in traditional medical systems, such as Ayurveda and traditional Chinese medicine (TCM) is particularly important. In Ayurveda, frankincense, known as “Shallaki” [*Boswellia serrata* Roxb. ex Colebr. (Burseraceae)], has been used for centuries to treat inflammatory conditions, joint disorders, and respiratory ailments (Gupta et al., 2011). Ayurvedic formulations often combine frankincense with other botanicals to enhance its therapeutic efficacy, particularly in managing rheumatoid arthritis (Amavata) and osteoarthritis (Siddiqui, 2011).

In Traditional Chinese Medicine (TCM), frankincense (“Ru Xiang”) is highly valued for its ability to promote blood circulation, reduce swelling, and alleviate pain (Efferth and Oesch, 2022). It is frequently used in combination with myrrh (“Mo Yao”), which is the gum resins obtained from trees of *Commiphora myrrha* (T.Nees) Engl. (Burseraceae) (Commiphor amyrrha Engl, 2025), to treat traumatic injuries, inflammation, and chronic pain conditions. TCM practitioners believe that frankincense can clear stagnant blood (“blood stasis”), making it a key metabolite in formulations for wound healing, menstrual disorders, and inflammatory diseases (Liao et al., 2021).

In African and Arabian traditional medicine, frankincense has played a crucial role in managing infections, wounds, and inflammatory conditions (Miran et al., 2022). In Oman and Somalia, for example, it is commonly used as a chewing resin for improving digestion and oral health, while its smoke is believed to have antiseptic and mood-enhancing properties (Khan and Rashan, 2025). Additionally, in Ethiopian traditional medicine, frankincense has been used in remedies for neurological disorders, including epilepsy and depression, highlighting its potential neuroprotective effects (Khalifa et al., 2023).

**FIGURE 1:**
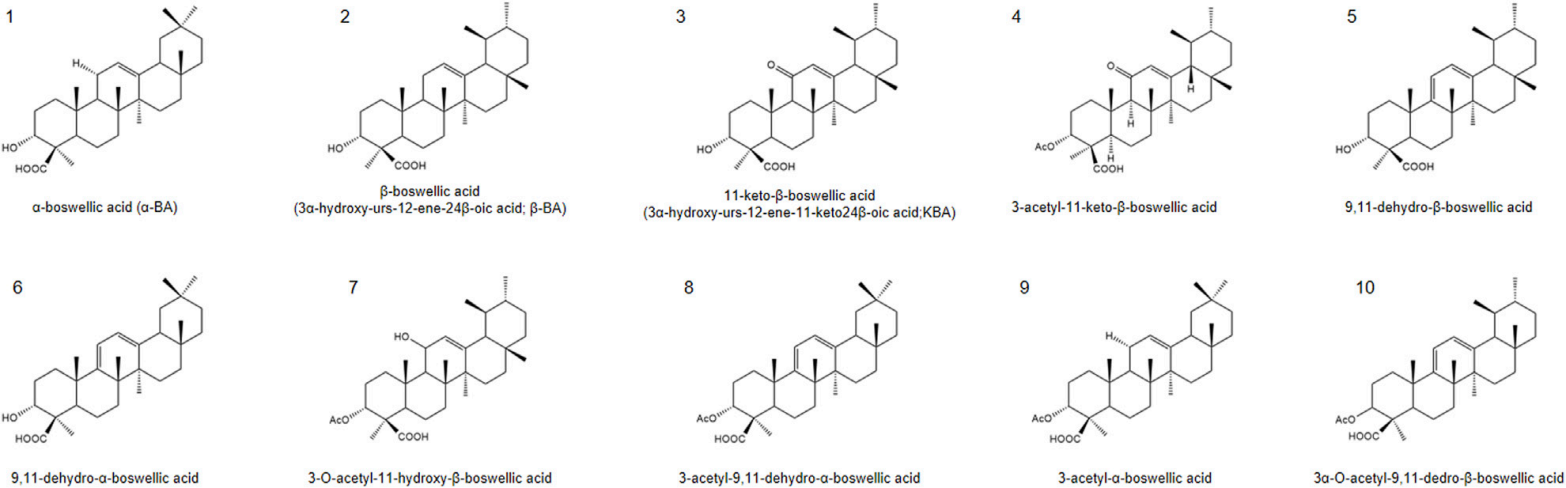
Chemical structures of representative boswellic acids: (1) α-boswellic acid, (2) β-boswellic acid, (3) 11-keto-β-boswellic acid (KBA), (4) 3-O-acetyl-11-keto-β-boswellic acid (AKBA), (5) 9,11-dehydro-β-boswellic acid, (6) 9,11-dehydro-α-boswellic acid, (7) 3-O-acetyl-11-hydroxy-β-boswellic acid, (8) 3-acetyl-9,11-dehydro-α-boswellic acid, (9) 3-acetyl-α-boswellic acid, (10) 3α-O-acetyl-9,11-dedro-β-boswellic acid.

In the past few decades, more than 200 natural products have been identified or isolated from frankincense, including terpenoids, polyphenols, essential oils, tannins, alkaloids, saponins and other metabolites (Efferth and Oesch, 2022; Hussain et al., 2022). Among these metabolites, terpenoids are the most abundant and predominant metabolites, with pentacyclic triterpenes receiving particular attention due to their notable biological activities. The primary active metabolites of frankincense are BAs with a pentacyclic triterpenoid structure (Huang et al., 2022), especially *α*-boswellic acid (*α*-BA), *β*-boswellic acid (*β*-BA), 11-keto-*β*-boswellic acid (KBA), 3-acetyl-11-keto-*β*-boswellic acid (AKBA), 9,11-dehydro-β-boswellic acid, 9,11-dehydro-α-boswellic acid,3-O-acetyl-11-hydroxy-β-boswellic acid, 3-acetyl-9,11-dehydro-α-boswellic acid, 3-acetyl-*α*-boswellic acid, 3α-O-acetyl-9,11-dedro-β-boswellic acid. The specific information is shown on the table (Figure 1).

Boswellic acids as a popular research subject, there are abundant related research reports on it, it also has been reviewed quite often. However, previous reviews on boswellic acids have primarily focused on their anti-inflammatory and pharmacological activities (Ammon, 2010; Börner et al., 2021). While these studies have provided valuable insights, they often lack systematic comparisons of pharmacokinetics across species, detailed evaluations of experimental design (e.g., dose ranges, active concentrations, control settings), and an in-depth analysis of translational limitations. In addition, the discussion of formulation strategies has mostly remained descriptive, with limited attention to advanced targeted delivery approaches.

In contrast, this review provides a comprehensive and updated synthesis by (1) highlighting species-specific metabolic and pharmacokinetic challenges, (2) critically appraising both *in vitro* and *in vivo* pharmacological studies with detailed methodological parameters, (3) integrating the latest nano formulation and ligand-targeted strategies for improving bioavailability, and (4) illustrating the diverse anti-inflammatory mechanisms of BAs through refined mechanistic diagrams and comparative tables. These features distinguish our work from previous reports and aim to bridge the gap between mechanistic studies and clinical translation.

## 2 Methods

This review adopted a systematic literature search strategy to ensure the inclusion of the most recent studies on boswellic acids and their delivery systems in anti-inflammatory therapy. The primary databases searched included PubMed, Web of Science, Scopus, and Embase. Additionally, Google Scholar was used as a supplementary source to identify potentially missed studies. To maintain the scientific rigor and reliability of the review, only peer-reviewed articles were included, while conference abstracts and unpublished theses or dissertations were excluded.

A combination of keywords and Boolean operators was employed during the search process to enhance both the precision and comprehensiveness of the retrieval. The primary search terms included but were not limited to “Boswellic acids,” “Boswellia extract,” and “Acetyl-11-keto-β-boswellic acid (AKBA),” in conjunction with inflammation-related terms such as “Anti-inflammatory,” “Inflammation,” “NF-κB,” and “5-lipoxygenase (5-LOX).” For studies focusing on delivery systems, additional keywords such as “Nanoformulation,” “Nanoparticles,” “Drug delivery,” and “Bioavailability enhancement” were incorporated to capture the latest applications of boswellic acids in drug delivery technologies. To improve the standardization of the search, controlled vocabulary terms were applied where appropriate, particularly in PubMed. For example, queries such as [“Boswellic Acids” (Title)] AND [“Anti-Inflammatory Agents” (Title)] were used to ensure the relevance of the retrieved literature. To ensure taxonomic accuracy, the nomenclature of all *Boswellia* species included in this review was verified using curated botanical databases, namely *Plants of the World Online* (POWO), and *World Flora Online* (WFO). Accepted names, author citations, this verification process ensured consistency in species identification across pharmacological and clinical literature.

The literature selection process followed strict inclusion and exclusion criteria. The inclusion criteria were as follows: (1) the study must be an *in vitro*, *in vivo*, or clinical trial, excluding purely computational models; (2) the research must focus on the anti-inflammatory properties of boswellic acids and their nano-delivery strategies rather than other pharmacological effects; (3) only English-language articles were considered to maintain consistency in academic communication. The exclusion criteria included review articles, conference abstracts, non-peer-reviewed studies, and those that were irrelevant to the primary topic.

## 3 Anti-inflammatory mechanisms of boswellic acid

**FIGURE 2:**
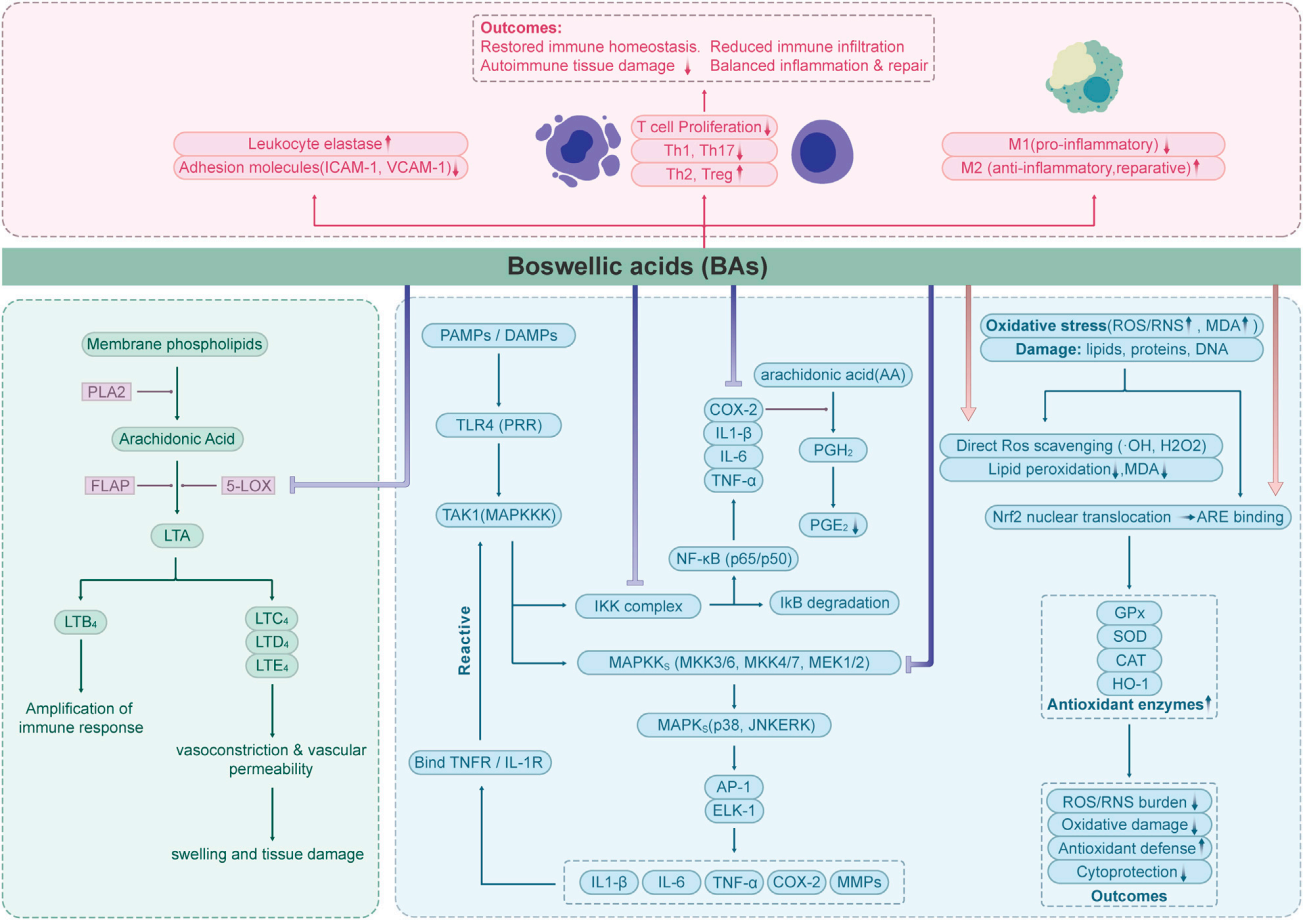
Flow chart depicting the effects of Boswellic acids (BAs) on immune and oxidative stress pathways.

Boswellic acids exhibit potent anti-inflammatory effects through multiple interconnected mechanisms, making it effective in managing inflammatory conditions. Here’s a structured breakdown of its key mechanisms (Figure 2).

### 3.1 5-LOX inhibition

The synthesis of leukotriene was inhibited by Inhibition of 5-Lipoxygenase (5-LOX) (Rådmark et al., 2007). 5-Lipoxygenase (EC 1.13.11.34) is the key enzyme catalyzing the initial steps of leukotriene biosynthesis and represents an important therapeutic target in inflammatory diseases. Inhibitors of 5-LOX are being actively investigated as potential anti-inflammatory agents (Lin et al., 2014; Gilbert et al., 2021).

Previous studies have confirmed that boswellic acids (BAs) act as specific non-redox inhibitors of 5-lipoxygenase (5-LOX) product formation. They exert their effects either by directly interacting with the 5-LOX enzyme or by blocking its translocation. Among the BAs, acetyl-11-keto-β-boswellic acid (AKBA) demonstrates the most potent inhibitory activity against 5-LOX products, with a reported IC_50_ value of 1.5 μM (in human neutrophils; non-redox, non-competitive; *in vitro*) (Safayhi et al., 1995). It directly inhibits 5-LOX by a selective, enzyme-directed, non-redox and non-competitive mechanism. β-Boswellic acid (β-BA) lacking the 11-keto function, only partially and incompletely inhibited 5-LOX (Sailer et al., 1996). Early studies on the pharmacological activity of boswellic acids (BAs) were primarily conducted *in vitro*, and the inhibitory effects observed varied across different experimental settings. According to the research and review by Werz, the efficacy of 5-LOX inhibitors in both intact cell and cell-free assays depends on assay conditions, such as substrate concentration, the presence of 5-LOX activating cofactors (e.g., phosphatidylcholine, lipid hydroperoxides, Ca^2+^), and the type of stimulus used to activate intact cells (Werz and Steinhilber, 2005; Werz and Steinhilber, 2006; Werz, 2007). In the study by Ulf Siemoneit et al., the inhibitory effects of different boswellic acids on 5-lipoxygenase (5-LOX) were evaluated across various *in vitro* test systems, including (1) purified 5-LOX enzyme, (2) supernatants of *E. coli* lysates, and (3) neutrophils. Both acetyl-11-keto-β-boswellic acid (AKBA) and 11-keto-β-boswellic acid (KBA) demonstrated strong inhibition of 5-LOX across all systems, while α-boswellic acid (α-BA) exhibited greater inhibitory activity than β-boswellic acid (β-BA). Notably, the study found that in the presence of albumin (10 mg/mL), the inhibitory effect of 11-keto boswellic acid (up to 30 μM) on neutrophil 5-LOX was abolished (Siemoneit et al., 2009). This finding explains why 11-keto boswellic acid (KBA) failed to inhibit 5-LOX product formation in human whole blood during *in vitro* assays, and why single oral administration of *Boswellia serrata Roxb. ex Colebr. (Burseraceae)* resin extract in a Phase I clinical trial did not suppress plasma leukotriene B4 levels in subjects. This may be attributed to the strong binding affinity of 11-keto boswellic acid to albumin (>95%) and its relatively low bioavailability *in vivo*, among other factors (as will be discussed in the “Pharmacokinetic and Metabolic Challenges of Boswellic Acids” section below).

### 3.2 Suppression of NF-κB pathway

The NF-κB signaling pathway regulates the expression of genes involved in immune responses, cell survival, and chronic inflammation. In its inactive state, NF-κB is sequestered in the cytoplasm through interaction with inhibitory proteins (IκBs). Upon stimulation—such as exposure to pro-inflammatory cytokines like TNF-α—signaling cascades are triggered that lead to the phosphorylation and subsequent degradation of IκBs. This allows NF-κB to translocate into the nucleus, where it initiates the transcription of pro-inflammatory genes (Hinz and Scheidereit, 2014).

This activation can be suppressed by blocking IκB kinase (IKK), thereby preventing IκB degradation. Acetyl-11-keto-β-boswellic acid (AKBA) has been shown to inhibit the NF-κB pathway, preventing IκBα phosphorylation and degradation, and reducing the nuclear translocation of NF-κB subunits (Syrovets et al., 2005a; Takada et al., 2006).

Acetyl-11-keto-β-boswellic acid (AKBA) has been shown to inhibit the NF-κB pathway, thereby reducing the expression of pro-inflammatory cytokines (e.g.: TNF-α, IL-1β, IL-6), chemokines, and inflammatory enzymes such as cyclooxygenase-2 (COX-2) and inducible nitric oxide synthase (iNOS) (shown in LPS-stimulated Mice model) (Cuaz-Pérolin et al., 2008). Experimental evidence from animal models of inflammatory bowel disease (IBD), psoriasis, osteoarthritis, and experimental autoimmune encephalomyelitis (EAE) has confirmed that AKBA-mediated suppression of the NF-κB pathway contributes to its anti-inflammatory effects (Wang et al., 2009; Nadeem et al., 2022; Zhou et al., 2022; Zhang and Jiang, 2023).

### 3.3 Antioxidant activity

Oxidative stress refers to a pathological condition in which the production of reactive oxygen species (ROS) and reactive nitrogen species (RNS) exceeds the capacity of the endogenous antioxidant defense system, leading to oxidative damage of cellular components such as lipids, proteins, and DNA (Jomova et al., 2023). Recent studies have demonstrated that boswellic acid exerts antioxidant effects through multiple mechanisms. One key mechanism is the direct scavenging of ROS—such as hydroxyl radicals (·OH) and hydrogen peroxide (H_2_O_2_)—via active functional groups like phenolic hydroxyls. This activity helps inhibit lipid peroxidation chain reactions and reduces the formation of oxidative end-products, such as malondialdehyde (MDA) (Liu and Ng, 2000; Barakat et al., 2018; Elgazar et al., 2023). In addition, boswellic acid can enhance the endogenous antioxidant defense system by promoting the nuclear translocation of nuclear factor erythroid 2–related factor 2 (Nrf2) (Upadhayay et al., 2022). Once translocated into the nucleus, Nrf2 binds to antioxidant response elements (AREs), leading to the transcriptional activation of various antioxidant enzymes, including glutathione peroxidase (GPx), superoxide dismutase (SOD), catalase (CAT), and heme oxygenase-1 (HO-1). This pathway significantly strengthens the cellular antioxidant capacity and protects against oxidative damage (Eltahir et al., 2020).

### 3.4 Immune cell modulation

Immune cell modulation refers to the process of regulating the function, activity, or population of immune cells—such as T cells, B cells, macrophages, and dendritic cells—through chemical, biological, or physical means, with the aim of either enhancing or suppressing immune responses (Strzelec et al., 2023). Boswellic acid has been shown to inhibit the activation of pro-inflammatory M1 macrophages while promoting the polarization toward the anti-inflammatory and tissue-reparative M2 phenotype. This shift contributes to the restoration of immune homeostasis by balancing inflammatory and reparative processes (Nischang et al., 2023; Wang et al., 2024). Moreover, studies have shown that boswellic acid can modulate T cell-mediated immune responses by inhibiting T cell proliferation and suppressing the differentiation of pro-inflammatory Th1 and Th17 cells, while promoting a shift toward anti-inflammatory Th2 responses. This immunomodulatory effect contributes to the attenuation of pathological damage in autoimmune diseases (Stürner et al., 2014; Meyiah et al., 2023). Additionally, it inhibits leukocyte elastase and adhesion molecule (ICAM-1, VCAM-1) expression, limiting immune cell infiltration into inflamed tissues (Safayhi et al., 1997; Roy et al., 2005).

### 3.5 Selective COX-2 inhibition

Unlike conventional nonsteroidal anti-inflammatory drugs (NSAIDs), boswellic acids have been proposed to preferentially inhibit COX-2 over COX-1 under certain conditions; however, several studies indicate that they may still exert inhibitory effects on COX-1 (e.g. 11-keto-β-boswellic acids inhibited COX-1 in stimulated human platelets, IC_50_ = 6–17 μM, with comparatively weaker inhibition of COX-2) (Siemoneit et al., 2008).

Boswellic acids (BAs) modulate prostaglandin biosynthesis mainly by downregulating COX-2 expression through suppression of NF-κB and AP-1 signaling, rather than by directly blocking the COX-2 catalytic site (Siemoneit et al., 2011; Verhoff et al., 2014). Compared to COX-1, BAs exhibit greater inhibitory potency against COX-2, as evidenced by lower IC_50_ values (Abdel-Tawab, 2021). This selective inhibition leads to a reduction in inflammation-associated prostaglandins such as PGE_2_, while preserving COX-1–mediated physiological functions like gastric mucosal protection, thereby minimizing the risk of gastrointestinal ulceration commonly associated with NSAID therapy.

### 3.6 Apoptosis induction in inflammatory cells

Resolution of Inflammation: AKBA induces apoptosis in hyperactive immune cells (e.g., macrophages, synoviocytes), curtailing prolonged inflammatory responses.

Boswellic acids can induce apoptosis by directly activating apoptosis-related caspase proteases, thereby initiating the intrinsic apoptotic program within cells (Takada et al., 2006; Kunnumakkara et al., 2018).

### 3.7 Decreased the expression of matrix metallopeptidase (MMPs)

Boswellic acids are reported to downregulate matrix metallopeptidases (MMPs), a family of zinc-dependent endopeptidases that play crucial roles in the degradation and remodeling of the extracellular matrix (ECM). Functionally, MMPs are involved in a variety of physiological and pathological processes, including wound healing, inflammatory responses, and tumor metastasis. By modulating MMP activity, boswellic acids may contribute to the regulation of tissue remodeling and inflammatory progression (Cui et al., 2017; de Almeida et al., 2022). In the context of inflammation, the overexpression of MMPs is closely associated with cartilage degradation, particularly in rheumatoid arthritis (RA) and osteoarthritis (OA). MMPs are considered potential biomarkers and key mediators of disease progression in these conditions (Visse and Nagase, 2003). To date, numerous phytochemicals have been investigated for their potential as matrix metalloproteinase (MMP) inhibitors (Alamgeer et al., 2020). Among these, boswellic acids—natural metabolites with potent anti-inflammatory properties—have attracted considerable attention for their ability to modulate MMP activity. Firstly, boswellic acids, especially acetyl-11-keto-β-boswellic acid (AKBA), are believed to directly interfere with the promoter activity of MMP genes. It has been demonstrated that AKBA downregulates the mRNA expression levels of MMP-2, MMP-3, and MMP-9 by suppressing their promoter activity, thereby reducing the synthesis of these matrix metalloproteinases (Ranzato et al., 2017; Zhang et al., 2020).

Moreover, current studies suggest that NF-κB is a key transcription factor responsible for the induction of MMP expression and is highly activated under inflammatory conditions. As discussed above, boswellic acids—particularly AKBA—suppress NF-κB activation by inhibiting the phosphorylation of IκBα, thereby preventing NF-κB nuclear translocation. This results in reduced transcriptional activation of MMP promoters, including those of MMP-2, MMP-3, and MMP-9 (Park et al., 2011). Moreover, experimental studies have demonstrated that inhibition of the mitogen-activated protein kinase (MAPK) signaling pathway—particularly in human fibroblasts, glioma cells, and macrophages under inflammatory stimulation—leads to a significant reduction in MMP-9 expression (Liang et al., 2012). AKBA has been shown to markedly inhibit the phosphorylation of p38 MAPK and ERK1/2, thereby suppressing MAPK-mediated transcriptional activation of MMPs(Syrovets et al., 2005b). These findings suggest that boswellic acids can reduce MMP expression not only through inhibition of NF-κB signaling but also via suppression of the MAPK cascade.

### 3.8 TLR and IFN pathways

In addition to classical anti-inflammatory mechanisms, boswellic acids (BAs) have been reported to modulate innate immune recognition pathways. AKBA markedly attenuated lipopolysaccharide (LPS)–induced cardiac dysfunction in mice, reducing NF-κB activation and pro-inflammatory cytokine release, thereby implicating suppression of Toll-like receptor 4 (TLR4)–mediated signaling (Taherzadeh et al., 2022). Such findings suggest that BAs may interfere with TLR-dependent activation cascades that drive inflammatory injury. Evidence also indicates that BAs affect interferon pathways. Several studies have documented reduced IFN-γ production in T-cell and macrophage models upon BA treatment, shifting immune responses away from Th1-dominated profiles (Gomaa et al., 2021; Moudgil and Venkatesha, 2022). Complementary *in silico* analyses predicted frankincense diterpenoids and triterpenoids to target IFN-γ and associated JAK/STAT components, further supporting the plausibility of BA–IFN pathway interactions (Halim et al., 2020). Nevertheless, not all reports are consistent: in certain macrophage models, BA failed to suppress IFN-γ–induced iNOS expression (Henkel et al., 2012), suggesting that modulation of interferon signaling may be context-dependent.

### 3.9 Structure-informed and computational insights into BA–target interactions

While extensive experimental studies have delineated the multiple signaling pathways regulated by boswellic acids, the question remains as to how these metabolites can engage such a wide array of molecular targets. Recent structural and computational investigations provide additional insights into this issue by examining their binding modes and dynamic interactions with inflammation-related proteins.

Recent computational and structural studies have started to rationalize how boswellic acids (BAs) engage multiple nodes in inflammatory pathways. For 5-lipoxygenase (5-LOX), Gaussian-accelerated MD (GaMD) combined with Markov state models (MSMs) captured stable AKBA-bound conformers and inhibitor transit routes, indicating a non-substrate pocket consistent with historical pharmacology (Liu et al., 2024). In parallel, MD simulations on AKBA derivatives reported persistent hydrogen-bond/hydrophobic networks and low RMSD/Rg fluctuations that align with their experimental inhibition trends, supporting a structure–activity rationale at 5-LOX (Bolbolian et al., 2020). At the enzyme level, natural-product structural work on 5-LOX highlights an exploitable allosteric region that can underlie isoform-selective inhibition—a logic that coheres with AKBA’s non-redox, non-competitive behavior (Gilbert et al., 2020). Classic biochemical evidence further anchors this model: AKBA directly targets 5-LOX at a pentacyclic-triterpene-selective site distinct from the arachidonate pocket, and photoaffinity labeling maps a regulatory site whose occupancy is modulated by triterpenes rather than competitive 5-LOX blockers (Safayhi et al., 1995; Sailer et al., 1998).

At the signaling level, an *in silico* focused analysis in psoriasis indicates frankincense diterpenoids/triterpenoids can engage cytokine hubs (TNF-α, IL-17/-13/-23/-36γ) and kinases (JAK1/2/3, MAPK2), offering a systems-level view that complements target-centric docking/MD (Halim et al., 2020). Meanwhile, matrix-facing evidence shows BAs dock to collagenase and elastase with supportive *in-vitro* inhibition, connecting BA chemistry to extracellular-matrix remodeling relevant to inflammatory skin biology (Hourfane et al., 2022). Finally, in osteoarthritic joint cells, β-BA integrates *in silico* predictions with proteomic/transcriptomic validation to suppress TLR4/IL-1R–driven innate responses and downstream NF-κB/MAPK axes, exemplifying a disease-context pipeline from computational prioritization to wet-lab confirmation (Franco-Trepat et al., 2023). In summary, structural and computational studies provide a mechanistic basis for the diverse anti-inflammatory actions of boswellic acids, while also highlighting open questions that warrant further biochemical validation.

Collectively, pharmacological evidence for boswellic acids has been derived from both *in vitro* systems (e.g., RAW264.7 macrophages, human PBMCs, microglia; 1–50 μM) and *in vivo* rodent models (25–200 mg/kg oral extracts for 1–4 weeks), typically with indomethacin or dexamethasone as positive controls. While these studies consistently demonstrate inhibition of NF-κB, NLRP3, and TLR signaling, variability in assay design, extract type, and duration underscores the importance of careful interpretation and the need for standardized clinical studies.

## 4 Anti-inflammatory effects of boswellic acids: evidence from experimental and clinical studies

In recent decades, both preclinical and clinical research on boswellic acids (BAs) has expanded substantially, driven by their potent anti-inflammatory potential. Numerous studies have explored the effects of BAs using a wide range of experimental systems—from cell-based assays to rodent disease models—alongside a growing number of clinical trials in patients with chronic inflammatory conditions. These studies have collectively advanced our understanding of pharmacological actions, mechanisms, and translational promise of BAs. The following sections provide a structured overview of the evidence base, beginning with mechanistic insights from pharmacological experiments.

### 4.1 Pharmacological study evidence

Due to the long-standing historical use of Boswellia extracts and the well-characterized bioactivity of their major metabolites—boswellic acids (BAs)—extensive pharmacological studies have been conducted to investigate their anti-inflammatory and immunomodulatory properties. A large body of literature provides mechanistic insights into the effects of boswellic acids through both *in vitro* and *in vivo* models.

Table 1 summarizes key findings from past research that illustrate the pharmacological activities of BAs and their underlying molecular mechanisms (Table 1).

**TABLE 1:** Preclinical pharmacological studies of boswellic acids in inflammatory and immune-related diseases.

Compound	Dose	Disease model	Controls/positive	Experimental design	Main outcomes	Proposed mechanism	References
Boswellic acids	100 and 200 mg/kg Boswellic acids (orally)	Cyclophosphamide-induced cystitis	MESNA (orally)	Cyclophosphamide injection to induce cystitis (rat)	bladder weight, edema, neutrophil infiltration, hemorrhage↓	MDA, CPO, NO, IL-6↓ TNF-α↓, CAT, GPx, SOD↑	Fatima et al. (2022)
AKBA	0,1, 5 μM AKBA	Autoimmune diseases incl. multiple sclerosis	No AKBA	CD4^+^ T cell differentiation assay	CD4 + T cells differentiate into Th17 cells ↓, and Th2 cells and Treg cells differentiate ↑	Th17 cells↓, IRAK1 signaling↓, pIRAK1↓, pSTAT3↓, inhibit IRAK1, STAT3, Ser727 phosphorylation	Stürner et al. (2014)
AKBA	10% AKBA (5, 10, 20, 40 mg/kg b.w) (Injection)	Inflammation and arthritis	2% AKBA (40 mg/kg) and diclofenac (10 mg/kg)	Carrageenan paw edema, FCA-induced arthritis (rat)	paw swelling, joint inflammation↓	5-LOX inhibition, NF-κB suppression, TNF, IL-1, IL-2, IL-4, IL-6, IFN↓	Banji et al. (2022)
AKBA	boswellic acid cream 10 mg/kg (oral or topical)	Osteoarthritis	ointment base without boswellic acid (oral or topical)	DMM model (mice)	cartilage erosion, synovitis, osteophyte formation↓ Inhibits inflammatory mediators and cartilage degradation	IL-1β, TLR signaling↓, 5-LOX↓	Wang et al. (2014a)
AKBA	2、4、8、16 or 32 μM AKBA for cell, 8 mg/kg AKBA for Animal (injection)	Synovitis in OA	0 μM AKBA for cell, 0.5 mL/kg saline for Animal (injection)	Primary rat FLS, ACLT + DMM OA model (rat)	FLS migration induced by LPS↓, Pain in the body, synovial inflammation and fibrosis ↓ Nrf2 nuclear translocation↑	ROS, IL-1β, IL-6, TNF-α, iNOS, COX-2, MMPs↓, Activates Nrf2/HO-1 pathway	Zhou et al. (2024)
LI13019F1 (AKBA, KBA, α-BA, β-BA, α-ABA, β-ABA)	75 mg/kg, 150 mg/kg, 300 mg/kg, LI13019F1 (orally)	Osteoarthritis	0.5% Carboxymethylcellulose Sodium (orally)	MIA-induced OA (rat)	weight bearing↑, Increased sensitivity to heat and mechanical stimulation↑, pain relief	IL-1β, IL-6, TNF-α, iNOS, COX-2, 5-LOX↓	Alluri et al. (2020)
Semi-synthetic acetoxyl-11-keto-β-boswellic acid (sAKBA)	sAKBA (5 mg·kg/day) (orally)	Colitis	dexamethasone (1 mg·kg/day) (orally)	3% DSS-induced colitis (mice)	leukocyte/platelet recruitment, P-selectin expression↓	5-LOX activity↓, CAM expression↓	Anthoni et al. (2006)
The boswellia extract H15	17.1 and 34.2 mg/kg daily H15, (orally) 3.4 and 5.1 mg/kg daily AKBA, (orally)	Ileitis	carriers NaHCO_3_ (subcutaneously) and tylose (orally)	Indomethacin-induced ileitis (rat)	leukocyte rolling/adhesion, tissue injury↓	5-LOX inhibition	Krieglstein et al. (2001)
Boswellic acids	*Boswellia. Serrata* extract 34.2 mg/kg/day (orally)	Colitis	Without *boswellia. Serrata* extract	Acetic acid-induced colitis (rat)	Antioxidant activity	LPO, inflammation↓, SOD↑, GPx↓, GSH↑	Hartmann et al. (2012)
α-BA	50 mg/kg, 100 mg/kg, 200 mg/kg α-BA (orally)	Gastric injury	100 mg/kg Cimetidine (orally)	Ethanol-induced gastric ulcer (rat)	gastric acidity, MDA↓	Nrf2/HO-1 activation, CAT, SOD, NO, PGE-2↑	Zhang et al. (2016)
Boswellic acids (BA-1 to BA-4)	50 mg–500 mg/kg Boswellic acids (orally)	Gastric ulcer	50 and 100 mg/kg cimetidine (orally)	Pylorus ligation, ethanol/HCl, ASA, indomethacin, heat stress (rat)	ulcer index, gastric acid↓, mucosal resistance, microcirculation↑	prostaglandin synthesis↑, leukotriene synthesis↓	Singh et al. (2008a)
Boswellic acids	125 and 250 mg/kg Boswellic acids (orally)	NAFLD	equal volumes carboxymethylcellulose (CMC) solution (orally)	High-fat diet-induced NAFLD (rat)	steatosis, liver index, insulin resistance, liver enzymes, TG↓	TNF-α, IL-6, iNOS, HNE↓, UCP-1, CPT-1↓	Zaitone et al. (2015)
Boswellic acids (65%)	125 and 250 mg/kg Boswellic acids (orally)	Parkinson’s disease	1:1 (*v*/*v*), dimethyl sulfoxide plus polyethylene glycol 400 vehicle	Rotenone-induced PD (rat)	Anti-inflammatory and neuroprotective	motor function, dopamine↑, IL-6, COX-2, TNF-α, NF-κB, 5-LOX↓	Ameen et al. (2017)
Boswellic acids	1.25, 2.5 and 3.75 mg/ear Boswellic acids (ointment)	Various acute/chronic inflammation	0.25 mg/ear piroxicam (ointment)	Ear edema, paw edema (mice), arthritis models (rat)	swelling, joint pain↓	Inhibit the synthesis of leukotrienes, 5-LOX ↓	Singh et al. (2008b)
Boswellic acids extract	1 g/kg Boswellic acids (orally)	Pulmonary fibrosis	receiving saline	Bleomycin-induced PF (rat), gamma irradiation	collagen deposition, inflammation↓, lung function↑	TGF-β1, TNF-α, lipid peroxidation↓, SOD, GSH-Px, 5-LOX ↓	Ali and Mansour (2011)
AKBA	25 mg/kg and 100 mg/kg Boswellic acids (orally)	Experimental autoimmune encephalomyelitis	No boswellic acids	EAE model (mice)	Immune modulation via T cell differentiation shift	IL-17, IFN-γ↓, TGF-β, GATA3, FoxP3↑, Th1/Th17↓, Treg↑	Shadab et al. (2024)

Upon treatment with either purified boswellic acids or Boswellia-derived mixtures containing BAs, several phenotypic changes have been consistently observed. In the immune system, these include reduced differentiation of inflammatory T effector cells, increased regulatory T cell differentiation, reduced leukocyte infiltration and systemic inflammatory markers, and diminished immune cell infiltration into inflamed tissues (Meyiah et al., 2023; Salama et al., 2023; Ragab et al., 2024). Concurrently, reduction of oxidative stress has also been reported, including decreased reactive oxygen species (ROS) formation, reactive nitrogen species (RNS), and lipid peroxidation products such as malondialdehyde (MDA) (Zaitone et al., 2015; Fatima et al., 2022). *In vivo* studies, notable attenuation of inflammation has been widely observed, including decreases in inflamed tissue weight, reduction of edema, and alleviation of inflammatory manifestations such as cartilage degradation, ulceration, attenuation of inflammation-associated manifestations such as tissue edema, cartilage degradation, and fibrosis. In diet-induced NASH models, BA treatment also mitigated organ degeneration, hepatic inflammation, and metabolic disturbances (Zaitone et al., 2015; Salama et al., 2023).

Based on these findings, we can conceptualize the primary mode of action of boswellic acids in anti-inflammatory effects: phenotypic changes—such as reduced inflammatory tissue edema, decreased oxidative stress, alleviated pain, and reduced inflammatory cell infiltration—are underpinned by molecular mechanisms including activation of antioxidant enzymes, inhibition of pro-inflammatory and hepatic enzymes, modulation of intracellular signaling pathways, and activation/inhibition of gene-regulating transcription factors. Pro-inflammatory cytokines are downregulated, and anti-inflammatory cytokines are upregulated. But most mechanistic data derive from *in vitro* systems, with activities strongly influenced by material type (purified AKBA/KBA vs. standardized extracts), controls (e.g., indomethacin, dexamethasone), and assay duration, which should be considered when extrapolating to disease relevance. Collectively, the preclinical pharmacological literature strongly supports the anti-inflammatory activity of boswellic acids *in vitro* and *in vivo*.

### 4.2 Clinical evidence and translational prospects

Although boswellic acids have demonstrated significant anti-inflammatory and immunomodulatory activities in both *in vitro* and *in vivo* studies, their real-world efficacy in humans requires further validation through clinical research. Given the complexity of disease microenvironments and individual variability, it remains uncertain whether boswellic acids possess strong translational potential in clinical settings. Therefore, it is necessary to systematically review and analyze the current clinical research to evaluate the therapeutic prospects and limitations of boswellic acids in the treatment of inflammatory and immune-related diseases. This section primarily focuses on randomized controlled trials (RCTs), which are considered the gold standard for assessing clinical efficacy due to their higher representativeness and methodological rigor.

Boswellic acids and their derivative formulations have shown promising therapeutic effects in various chronic inflammatory conditions. In multiple randomized, double-blind, placebo-controlled trials involving osteoarthritis (OA) patients, several Boswellia serrata Roxb. ex Colebr. (Burseraceae)–derived formulations enriched in boswellic acids—such as Boswel^®^, Aflapin^®^, and 5-Loxin^®^—were found to significantly improve pain scores, joint stiffness, and functional impairment. Some formulations also demonstrated the ability to reduce inflammatory markers (e.g., TNF-α, IL-6, CRP) and matrix metalloproteinases (MMPs) (Sengupta et al., 2008; Vishal et al., 2011; Kizhakkedath, 2013). In dermatological conditions such as eczema and psoriasis, boswellic acid-based treatments were reported to alleviate erythema, scaling, and pruritus with good tolerability (Togni et al., 2014). In patients with inflammatory bowel diseases (IBD), including Crohn’s disease and chronic colitis, Boswellia extracts significantly improved clinical scores and remission rates, suggesting modulatory effects on intestinal mucosal inflammation (Gerhardt et al., 2001; Gupta et al., 2001). However, some RCTs have shown no statistically significant differences between treatment and placebo groups in patients with Crohn’s disease or collagenous colitis (Madisch et al., 2007; Holtmeier et al., 2011) (Table 2).

**TABLE 2:** Clinical trials of boswellic acids and formulations in inflammatory and chronic diseases.

Formulation/compound	Disease	Study design	Sample size/duration	Main outcomes	References or trial id
Bosexil® (Phytosome® boswellic acids formulation)	Erythematous eczema, psoriasis	RCT, double-blind, placebo-controlled	59 subjects, 30 days	70% improvement in scaling, 50% improvement in erythema, no worsening, placebo: 90% no improvement, 10% worsening	Togni et al. (2014)
Curcumin + boswellic acids	Knee osteoarthritis	RCT, double-blind, placebo-controlled	201 subjects, 12 weeks	Pain relief	Haroyan et al. (2018)
Boswellin® (AKBA + βBA)	Knee osteoarthritis	RCT, double-blind, placebo-controlled	48 subjects, 120 days	Pain↓, stiffness↓, knee function↑, joint space↑, osteophytes↓	Majeed et al. (2019)
Aflapin® (30% AKBA)	Knee osteoarthritis	RCT, double-blind, placebo-controlled	60 subjects, 30 days	Improved VAS, LFI, WOMAC, TNF-α, CRP, MMP-3 decreased	Vishal et al. (2011)
5-Loxin® (30% AKBA)	Knee osteoarthritis	RCT, double-blind, placebo-controlled	75 subjects, 90 days	Improved VAS, LFI, WOMAC, TNF-α, IL-1β, IL-6, CRP, MMP-3 decreased	Sengupta et al. (2008)
MSM + boswellic acids	Knee osteoarthritis	RCT, interventional, supplement	200 subjects, 60 days	Improved VAS and Lequesne Index in 2 and 6 months, reduced NSAID use	Notarnicola et al. (2011)
BOSWELAN	Multiple sclerosis	Phase IIa, open-label	29 subjects, 8 months	MRI cortical lesion volume changes	Faizy et al. (2019)
H15 Boswellia extract	Crohn’s disease	RCT, double-blind, parallel group	102 subjects, 8 weeks	CDAI reduced by 90 points vs. mesalazine 53 points	Gerhardt et al. (2001)
*Boswellia serrata* gum resin	Bronchial asthma	RCT, double-blind, placebo-controlled	40 subjects, 6 weeks	Improved respiratory symptoms, FEV1, FVC, PEFR increased	Gupta et al. (1998)
*Boswellia serrata* gum resin	Chronic colitis	RCT, double-blind, placebo-controlled	30 subjects, 6 weeks	14/20 patients achieved remission	Gupta et al. (2001)

Beyond the conditions mentioned above, boswellic acids have also demonstrated neuroprotective potential in central nervous system disorders such as multiple sclerosis (MS), where MRI findings indicated a reduction in cortical lesion progression (Stürner et al., 2018). In respiratory diseases such as bronchial asthma, Boswellia extracts were associated with improvements in pulmonary function and a reduction in acute exacerbations (Gupta et al., 1997). Notably, even in non-typical indications such as kidney stones, a reduction in stone volume was observed following intervention (Al-Marhoon et al., 2023). Some studies have also suggested that combining boswellic acids with other botanicals, such as curcumin, may result in synergistic anti-inflammatory effects (Sethi et al., 2022). Collectively, preclinical and early-phase clinical trials support the therapeutic potential and favorable safety profile of boswellic acids in various inflammatory disorders, though their long-term efficacy and mechanisms of action require further systematic investigation.

At present, clinical research specifically targeting boswellic acids remains limited and relatively underdeveloped. While the focus of this review is on boswellic acids (BAs), most published RCTs have investigated *Boswellia serrata Roxb. ex Colebr. (Burseraceae)* extracts—such as H15 extract, Boswelan^®^, or native resin capsules—as the intervention. These extracts often contain a complex mixture of multiple boswellic acids along with various resinous and volatile metabolites. However, the precise content of boswellic acids is frequently not standardized, nor is it specified which particular BA (e.g., AKBA, KBA) is responsible for the observed activity. Many RCTs only refer to “standardized Boswellia extract” without indicating the identity or percentage of active metabolites such as AKBA (typically expected to range from 3% to 10%) (Börner et al., 2021; Majeed et al., 2021). This lack of specificity makes it difficult to attribute observed therapeutic effects to boswellic acids alone, as synergistic contributions from other metabolites cannot be excluded. Only a few studies have employed purified boswellic acid monomers (e.g., AKBA) as the sole active metabolite. Therefore, existing clinical evidence is insufficient to directly validate the standalone clinical efficacy of boswellic acids, leading to a clear disconnect between mechanistic pharmacological studies and clinical applications.

## 5 Toxicological and safety profile of boswellic acids

In addition to their pharmacological efficacy, the toxicological and safety characteristics of boswellic acids (BAs) must be carefully considered to provide a balanced overview. Both preclinical toxicology data and clinical adverse drug reaction (ADR) profiles are summarized below.

### 5.1 Preclinical toxicology

Animal studies indicate that Boswellia extracts and isolated boswellic acids have relatively low acute toxicity. Reported oral LD_50_ values in rodents exceed 2,000 mg/kg, suggesting low acute lethality (Efferth and Oesch, 2022). Subacute and subchronic toxicity studies showed that repeated administration of standardized *Boswellia serrata Roxb. ex Colebr. (Burseraceae)* extracts (up to 1,000 mg/kg/day) in rats produced no major organ toxicity, although mild hepatic enzyme elevations and gastrointestinal irritation were occasionally observed (Ammon, 2010). Limited animal data, including small-scale supplementation studies in dogs, suggest overall good tolerability, though comprehensive long-term controlled toxicity studies in canines are lacking (Siddiqui, 2011).

### 5.2 Clinical safety and adverse events

In randomized controlled trials of osteoarthritis patients, standardized Boswellia extracts such as 5-Loxin^®^ and Aflapin^®^ were generally safe, with most adverse events being mild gastrointestinal symptoms (e.g., nausea, diarrhea, abdominal pain) and occurring at rates comparable to placebo (Sengupta et al., 2008; Vishal et al., 2011; Kizhakkedath, 2013). Dermatological applications in eczema and psoriasis reported good tolerability, with occasional mild erythema or pruritus, and no serious events (Togni et al., 2014). Trials in inflammatory bowel disease indicated that Boswellia extracts were not associated with severe ADRs, although mild gastrointestinal complaints were again the most common side effects (Gerhardt et al., 2001; Holtmeier et al., 2011). In patients with multiple sclerosis, *Boswellia* supplementation was well tolerated with no serious adverse events or clinical evidence of hepatic or renal toxicity (Stürner et al., 2018).

Overall, boswellic acids exhibit a favorable toxicological and safety profile across preclinical and clinical studies. Acute and sub chronic toxicity appear low, and adverse reactions are typically limited to mild gastrointestinal or transient dermatologic effects. Serious toxicity has not been reported to date, though comprehensive long-term and reproductive or genotoxicity studies remain limited.

## 6 Pharmacokinetic and metabolic challenges of boswellic acids

Among the various types of boswellic acids (BAs), 11-keto-β-boswellic acid (KBA) and 3-acetyl-11-keto-β-boswellic acid (AKBA) are considered the most pharmacologically active (Du et al., 2015). However, their absorption in the human body is influenced by multiple molecular and physiological factors. One of the key elements affecting the absorption of BAs is their solubility, which is closely related to their chemical nature (Cui et al., 2024). In the digestive system, substances that are soluble in aqueous environments have a higher likelihood of being absorbed because they can dissolve in gastrointestinal fluids. However, boswellic acids are inherently steroid-like molecules with lipophilic properties, resulting in low aqueous solubility and poor dissolution in intestinal fluids (Du et al., 2015). According to a study by Phillip Krüger, the Caco-2 apparent permeability coefficient (P_app) of KBA at 37 °C was determined to be 1.69 × 10^−6^ cm/s. Under the same experimental conditions, AKBA exhibited a permeability of less than 0.05%, and it was not possible to determine a precise P_app value for AKBA (Krüger et al., 2008). These findings suggest that KBA possesses moderate permeability, whereas AKBA exhibits poor permeability.

In addition to solubility and permeability, the oral bioavailability of these metabolites also depends significantly on their distribution and metabolic characteristics (Hill et al., 2017).

### 6.1 Tissue distribution and target organ accumulation of boswellic acids

Boswellic acids (BAs)—primarily 11-keto-β-boswellic acid (KBA) and 3-O-acetyl-11-keto-β-boswellic acid (AKBA)—exhibit a distinct *in vivo* distribution profile that is influenced by their lipophilic nature and inherent challenges with oral bioavailability (Table 3).

**TABLE 3:** Pharmacokinetics and tissue distribution of boswellic acids in human and animal studies.

Dosage	Methods	Concentrations of BAs determined in plasma (µM) or brain* (ng/g)								
		β-BA	α-BA	β-ABA	α-ABA	KBA	AKBA	Sample type	Population	References
1,600 mg/day	HPLC^a,h^	NA	NA	NA	NA	1.7	NA	Plasma	Human (n = 1)	Tawab et al. (2001)
333 mg/day	HPLC^b,h^	NA	NA	NA	NA	2.72 ± 0.18	ND	Plasma	Human (n = 12)	Sharma et al. (2004)
4 × 786 mg/day	HPLC^c^	10.1	3.5	2.4	4	0.3	0.1	Plasma	Human (n = 1)	Büchele and Simmet (2003)
3 × 800 mg/day	HPLC-ESI/MS^b,e^	6.35 ± 0.52	NA	4.9 ± 0.5	NA	0.3 ± 0.1	0.04 ± 0.01	Plasma	Human (n = 3)	Tausch et al. (2009)
4,200 mg/day	HPLC-ESI/MS^d^	(0.19–26.20)	(0.08–10.59)	(0.26–12.31)	(0.14–5.99)	(0.01–0.52)	(0–0.03)	Plasma	Human (n = 14)	Gerbeth et al. (2011)
500 mg/day	HPTLC^d^	NA	NA	NA	NA	NA	(0.05–0.13)	Plasma	Human (n = 6)	Shah et al. (2007)
800 mg/day (Micellar BSE)	HPLC-MS/MS^d,h^	NA	NA	NA	NA	0.420 (0.499–4.122)	0.007 (0.130–4.311)	Plasma	Human (n = 20)	Schmiech et al. (2024)
800 mg/day (Native BSE)	HPLC-MS/MS^d,h^	NA	NA	NA	NA	0.058 (0.150–4.610)	0.002 (0.014–0.552)	Plasma	Human (n = 20)	
240 mg/kg	HPLC-ESI/MS^f,h^	2.33 ± 1.02	1.19 ± 0.63	1.65 ± 0.11	0.48 ± 0.06	0.38 ± 0.15	0.33 ± 0.16	Plasma	Rats (n = 3)	Gerbeth et al. (2013)
240 mg/kg	HPLC-ESI/MS^g^	1,066.6	485.1	163.7	43	11.6	37.5	Brain	Rats (n = 6)	
240 mg/kg	HPLC-APCI/MS^h^	NA	NA	NA	NA	0.4	0.2	Brain	Rats (n = 9)	Reising et al. (2005)
240 mg/kg	HPLC-APCI/MS^h^	NA	NA	NA	NA	99	95	Brain	Rats (n = 9)	
333 mg/day	LC-MS/MS^f,h^	NA	NA	NA	NA	0.051 ± 0.009	0.016 ± 0.003	Plasma	Human (n = 10)	Kulkarni et al. (2021)
450 mg/kg	UHPLC-LTQ-Orbitrap-MS^f,h^	NA	NA	NA	NA	0.145 ± 0.033	0.414 ± 0.006	Plasma	Rats (n = 6)	Fan et al. (2023)
450 mg/kg	UHPLC-LTQ-Orbitrap-MS^g,h^	NA	NA	NA	NA	0.003	0.360 ± 0.103	Plasma	Rats (n = 6)	
128 mg/kg (Nat-BE)	LC-MS/MSf^,h^	NA	NA	NA	NA	ND	0.074 ± 0.117	Plasma	Rats (n = 6)	Meins et al. (2018)
128 mg/kg (Sol-BE)	LC-MS/MSf^,h^					0.934 ± 0.176	1.851 ± 0.181	Plasma	Rats (n = 6)	
21.45 mg/kg KBA + 46.88 mg/kg AKBA, (BSE – Normal)	HPLC-ESI/MS^f,h^	NA	NA	NA	NA	0.600 ± 0.213	0.701 ± 0.162	Plasma	Rats (n = 6)	Wang et al. (2014b)
21.45 mg/kg KBA + 46.88 mg/kg AKBA, (BSE – Arthritic)	HPLC-ESI/MS^f,h^	NA	NA	NA	NA	0.198 ± 0.084	0.228 ± 0.075	Plasma	Rats (n = 6)	
21.45 mg/kg KBA + 46.88 mg/kg AKBA, (HLXLD – Normal)	HPLC-ESI/MS^f,h^	NA	NA	NA	NA	2.081 ± 0.661	2.805 ± 0.373	Plasma	Rats (n = 6)	
21.45 mg/kg KBA + 46.88 mg/kg AKBA, (HLXLD – Arthritic)	HPLC-ESI/MS^f,h^	NA	NA	NA	NA	0.366 ± 0.100	0.937 ± 0.261	Plasma	Rats (n = 6)	
171 mg/kg (Nanoformulation)	LC-MS/MS^g,h^	NA	NA	NA	NA	NA	0.652	Plasma	Rats (n = 6)	Truzzi et al. (2025)
171 mg/kg (native extract)	LC-MS/MS^g,h^	NA	NA	NA	NA	NA	0.088	Plasma	Rats (n = 6)	

NA, not analyzed, ND, not detectable, a = approximation, b = mean ± SE, c = absolute contents, d = [range], e = steady state concentration, f = mean ± SD, g = mean, h = Cmax.

In animal studies, following a single oral dose of *Boswellia serrata Roxb. ex Colebr. (Burseraceae)* extract, both KBA and AKBA have been detected in plasma as well as in brain tissue. For example, in a rat model reported by Reising et al. (2005), KBA reached concentrations of approximately 99 ng/g in brain tissue (which corresponds to roughly 9.9 ng/mL when expressed in brain homogenate), while AKBA was found at about 95 ng/g. In these animals, plasma levels were considerably higher, with KBA generally ranging between 150 and 200 ng/mL and AKBA present at somewhat lower levels (Reising et al., 2005).

Human studies using solid lipid formulation designed to enhance the bioavailability of Bas. In the study of Kulkarni et al. (2021), peak plasma concentrations were reached at about 1.5 h for AKBA and 2.3 h for KBA, with C_max values of approximately 8.04 ng/mL for AKBA and 23.83 ng/mL for KBA. The elimination half-life was notably longer for AKBA (around 6.8 h) compared to KBA (approximately 2.45 h). These relatively low plasma levels reflect both species differences and formulation strategies aimed at improving bioavailability (Kulkarni et al., 2021).

Furthermore, a study by Fan et al. (2023) in rats demonstrated that when Boswellia extract is administered in combination with myrrh (a compatibility that is often used in traditional formulations), the plasma C_max values of both metabolites are modulated. In that setting, AKBA reached a plasma C_max of around 212 ng/mL before compatibility, with both AKBA and KBA levels declining upon co-administration with myrrh (Fan et al., 2023).

Together, these findings highlight that although BAs have inherently low oral bioavailability, they do distribute into key compartments, including the central nervous system, as evidenced by their measurable concentrations in brain tissue at the nanogram-per-gram level—and circulate systemically at concentrations that may be pharmacologically relevant for anti-inflammatory, analgesic, and anticancer effects. Notably, the lipophilic nature of boswellic acids enables their accumulation in tissues such as the liver and brain, despite their overall low plasma concentrations. Reising et al. (2005) detected KBA and AKBA in rat brain tissue at levels up to ∼100 ng/g, following oral administration of *Boswellia serrata Roxb. ex Colebr. (Burseraceae)* extract (Reising et al., 2005). These tissue distributions are critical for the therapeutic potential of boswellic acids in conditions such as neuroinflammation and arthritis.

### 6.2 Metabolic pathways and biotransformation of boswellic acids

According to the study conducted by Philipp Krüger et al., KBA (11-keto-β-boswellic acid) undergoes extensive phase I metabolism in both rat liver microsomes, hepatocytes, and human liver microsomes. The predominant metabolic pathway involves oxidation to hydroxylated metabolites, which appears to be the main route of KBA biotransformation *in vivo*. In parallel, the study reported a similar metabolic profile of KBA in in vitro experiments using rat plasma and liver tissue, indicating good correlation between *in vitro* and *in vivo* metabolic behavior (Krüger et al., 2008).

Importantly, the study did not detect any metabolites of AKBA (3-O-acetyl-11-keto-β-boswellic acid) *in vivo* and thus refuted the previously assumed hypothesis that AKBA is deacetylated to form KBA *in vivo*. This finding suggests that AKBA may exhibit poor bioavailability and metabolic stability under physiological conditions, highlighting the necessity for further investigation into its pharmacokinetics and delivery strategies (Krüger et al., 2008). In contrast to the findings of Philipp Krüger et al., a study published by Cui et al. (2016) provided more detailed insight into the phase I and phase II metabolism of both AKBA (3-O-acetyl-11-keto-β-boswellic acid) and KBA (11-keto-β-boswellic acid) in human liver microsomes (HLM) and human intestinal microsomes (HIM). This study demonstrated that deacetylation is the initial metabolic step for AKBA, primarily catalyzed by carboxylesterase 2 (CE2). Subsequently, KBA becomes the predominant form found in human plasma (Cui et al., 2016).

According to Cui et al. (2016), KBA is metabolized via CYP-mediated oxidation in human microsomes, with CYP3A4, CYP3A5, and CYP3A7 all capable of catalyzing hydroxylation of KBA; among these, CYP3A4 plays a predominant role in forming mono-hydroxylated KBA metabolites (e.g. 21- and 20-mono-hydroxylated KBA) in human liver preparations (Cui et al., 2016). To reconcile the discrepancies with Krüger’s findings, Cui et al. proposed that AKBA deacetylation may be a human-specific reaction, and not observable in rodents or other species. This hypothesis is supported by their cross-species comparative analysis, which revealed significant interspecies differences in both the presence of deacetylation activity and the distribution of hydroxylated KBA metabolites. Specifically, the deacetylation of AKBA was only observed in human tissues, while it was absent in six other animal species studied. This suggests that AKBA metabolism exhibits pronounced species selectivity and highlights the limitations of extrapolating metabolic profiles from animal models to humans (Cui et al., 2016).

To date, the metabolic studies on boswellic acids have primarily focused on Phase I biotransformation. According to Krüger et al. (2008), KBA (m/z 469.4 [M−H] ^-^, t = 11.3 min) undergoes extensive oxidation, resulting in at least three monohydroxylated metabolites (m/z +16), six dihydroxylated metabolites (m/z +32), and two monohydroxylated-dehydrogenated metabolites (m/z +14). Similarly, AKBA (m/z 511.4 [M−H] ^-^, t = 12.2 min) was shown to produce at least three monohydroxylated metabolites (Krüger et al., 2008). Further structural elucidation by Cui et al. (2016) using nuclear magnetic resonance (NMR) and tandem mass spectrometry (MS/MS) confirmed the identity of four major KBA metabolites: (1) 21-β-hydroxy-11-keto-β-boswellic acid (2) 20-β-hydroxy-11-keto-β-boswellic acid (3) 16-β-hydroxy-11-keto-β-boswellic acid (4) 30-hydroxy-11-keto-β-boswellic acid These findings suggest that hydroxylation at multiple positions is the dominant metabolic pathway for KBA, reflecting a diverse pattern of CYP-mediated oxidation (Cui et al., 2016).

In terms of metabolic stability, data from Sharma and Jana (2020) demonstrated that boswellic acids exhibit limited stability in simulated gastric and intestinal fluids, as well as in intestinal S9 fractions, raising concerns about their oral bioavailability (Sharma and Jana, 2020). Importantly, recent pharmacokinetic profiling indicates that neither AKBA nor KBA undergo Phase II conjugation reactions, such as glucuronidation or sulfation, as evidenced by the study of Rajabian et al. (2023). This absence of Phase II metabolism further emphasizes the predominant role of oxidative Phase I pathways in boswellic acid clearance (Rajabian et al., 2023).

## 7 Strategies to enhance the bioavailability of boswellic acids

Numerous pre-clinical pharmacological and clinical studies have demonstrated that boswellic acids (BAs) possess significant anti-inflammatory activity. However, the strong hydrophobicity and poor water solubility of BAs result in extremely low oral absorption (Sharma et al., 2010). Pharmacokinetic studies have shown that the systemic absorption of BAs—particularly KBA and AKBA—is very limited in both animals and humans (Hüsch et al., 2013).

Several Boswellia-derived formulations are already available on the market or have been tested in clinical settings. These include Casperome™ (Phytosome^®^ technology), which improves BA solubility and systemic absorption (Hüsch et al., 2013), an AKBA-enriched extract evaluated in osteoarthritis trials and shown to be safe and effective in reducing joint pain (Sengupta et al., 2008), a next-generation extract with enhanced bioavailability and anti-inflammatory efficacy (Vishal et al., 2011) and a topical formulation used in dermatological conditions such as eczema and psoriasis (Togni et al., 2014).

Despite these advances, important limitations remain. Most current formulations rely on oral or topical routes, and although they improve absorption compared to raw extracts, the overall bioavailability of boswellic acids is still relatively low. In addition, most clinical studies have been of short duration and modest sample size, and long-term safety or pharmacokinetic consistency data are lacking. These constraints highlight that while current formulations provide proof-of-concept for clinical use, further optimization is required. Given the limited oral bioavailability of BAs observed in clinical studies, optimization strategies have gained increasing attention. To address these challenges, various drug delivery strategies and formulation technologies have been developed to enhance the solubility, permeability, and bioavailability of BAs. This section summarizes the major improvement approaches and the supporting experimental evidence (Table 4).

**TABLE 4:** Drug delivery strategies to improve the solubility and bioavailability of boswellic acids.

Delivery strategy	Description	Improvement	Study stage	References
Phospholipid complex (Phytosome)	Complexation of BAs with phosphatidylcholine to enhance lipophilicity and membrane permeability	Solubility and absorption significantly increased, *in vitro* and *in vivo* studies showed higher uptake than free BAs	Animal/*in vitro*	Sharma et al. (2010)
Phospholipid nanocluster (Naturosomes)	BA-loaded phospholipid nanoclusters prepared by spray drying to improve dispersion and permeability	Solubility ↑ ∼16-fold, dissolution from ∼31% to >99%, permeability ↑ to ∼79% vs. ∼20% for free BAs	Animal/*in* *vitro*/permeability assay	Usapkar et al. (2024)
Self-Nanoemulsifying Drug Delivery System (SNEDDS)	Oil–surfactant–co-emulsifier blend forming Nanoemulsions upon dilution, improving dissolution and oral absorption	Solubility of 11-keto-β-boswellic acid (KBA) and 3-O-acetyl-11-keto-β-boswellic acid (AKBA) ↑ 2.7-fold and 2.3-fold, Cmax ↑ ∼2.0-fold, AUC ↑ ∼2.0-fold (*in vivo*)	Animal (oral)	Ting et al. (2018)
Micellar solubilization	Water-soluble micelles delivering BAs as microemulsions for enhanced gastrointestinal uptake	AKBA AUC ↑ ∼56-fold, Cmax ↑ ∼25-fold, improved bioavailability for all BA metabolites	Animal (oral)	Meins et al. (2018)
Solid dispersion/lipid-based particles	Lipid-based solid dispersions or sustained-release particles to improve solubility and control release	Solubility and dissolution ↑ ∼5-fold, improved pharmacokinetic (PK) parameters vs. free BAs	Animal (oral)	Mishra et al. (2022)
Polymeric nanoparticles (e.g., PLGA)	Encapsulation of AKBA in poly (lactic-co-glycolic acid) (PLGA) nanoparticles for stability and controlled release	Cmax ↑ ∼6-fold, AUC ↑ ∼9-fold, enhanced anti-inflammatory effect *in vivo*	Animal (oral)	Bairwa and Jachak (2015), 2016
Liposome formulation	Phospholipid vesicles encapsulate BAs to improve dispersibility and tissue targeting	Increased plasma concentration and prolonged circulation, improved anti-inflammatory efficacy	Animal (oral)	Majeed et al. (2019)
Cyclodextrin inclusion complex	Complexation of BAs with cyclodextrins to enhance aqueous solubility and dissolution	Significant increase in dissolution rate and *in vitro* permeability, improved oral bioavailability *in vivo*	Animal (oral)	Vijayarani et al. (2020)
Proniosome	Proniosomal gel: Preparation of nonionic surfactant vesicle gel	The *in vitro* transdermal test showed that the cumulative transdermal volume within 24 h was 84.8 mg/cm^2^	Animal/*in vitro*	Mehta et al. (2016)
Nanoemulsions	Transdermal Nanoemulsion system: Nonionic surfactants/PEG form water-in-oil microemulsions with particle sizes <100 nm, Nano-emulsion hydrogel: Nano-emulsions containing vegetable oil and surfactants for preparing gel matrices	The *in vitro* permeability was higher than that of the control, and the inhibition rate in the *in vivo* inflammation test was better than that of piroxicam. The transdermal flux of nano-emulsion gel is approximately 3.25 times that of ordinary gel	Animal/*in vitro*/*in* *vivo*	Gohel et al. (2015), Mostafa et al. (2015)

### 7.1 Anoparticle carriers (polymeric nanoparticles)

Polymeric nanoparticles such as PLGA significantly improve the oral absorption of BAs. In a study by Bairwa et al., PLGA nanoparticles loaded with KBA increased its oral bioavailability by approximately 7-fold (Bairwa and Jachak, 2016); In a subsequent study, AKBA-loaded PLGA nanoparticles enhanced its C_max by about 6-fold and AUC by 9-fold. These nanoparticles also showed enhanced anti-inflammatory activity in a rat model of CAR-induced inflammation (Bairwa and Jachak, 2015). Similarly, natural polymer-based nanoparticles such as chit osan have also been used to deliver BAs, improving their anti-inflammatory and antioxidant effects in the nervous system (Bairwa and Jachak, 2015; Ding et al., 2016). Overall, polymeric nanoparticles markedly increase drug concentrations and efficacy, although studies remain at the animal experimental stage.

### 7.2 Solid dispersions

Solid dispersions improve BA solubility and dissolution rate by co-melting or co-dissolving BAs with hydrophilic polymers. A study showed that solid dispersions of BAs prepared with polyethylene glycol PXM 188 and 407 at ratios of 1:2 (PXM188) and 1:1 (PXM407) demonstrated optimal performance in saturation solubility and *in vitro* release studies (Tambe et al., 2018). This system significantly enhances the water solubility and dissolution of BAs and offers a simple and practical oral delivery route. However, it currently lacks vivo absorption data.

### 7.3 Self-nanoemulsifying drug delivery systems (SNEDDS/SNES)

SNEDDS enhances BA solubilization by spontaneously forming nanoemulsions from oil and surfactants. According to Ting et al., the SNES formulation increased the aqueous solubility of KBA and AKBA by approximately 2.7-fold and 2.3-fold, respectively. In rabbit pharmacokinetic studies, the formulation improved the bioavailability of KBA and AKBA by about 2.2-fold and 2.0-fold compared to standard oil suspensions (Ting et al., 2018).These findings indicate that SNEDDS can stabilize BA particles and enhance GI absorption, demonstrating promising results for future clinical application.

### 7.4 Mplexes/phytosomes (phytosome)

Complexing BAs with phospholipids markedly improves hydrophilicity and membrane permeability. Hüsch et al. showed that formulating standardized Boswellia extract with soybean phospholipids into Casperome™ increased the AUC of KBA by approximately 7-fold and β-BA by 3-fold in mice. Notably, Casperome™ elevated KBA and AKBA concentrations in brain tissue by nearly 35-fold (Hüsch et al., 2013). Similarly, the novel “naturosome” formulation increased BA water solubility by 16-fold, *in vitro* cumulative release from ∼31% to >99%, and permeability from ∼20% to ∼79% (Usapkar et al., 2024). Early *in vitro* models have also confirmed that BA–phosphatidylcholine complexes achieve better intestinal absorption compared to free BA (Sharma et al., 2010). These results suggest that phospholipid complexes significantly improve BA absorption and distribution, with some formulations already moving toward clinical development (Hüsch et al., 2013).

### 7.5 Liposomes and elastic vesicles (spanlastics, proniosomes)

For topical delivery, lipid vesicles can enhance skin permeation. Badria et al. reported that a Spanlastic nanovesicle formulation of AKBA (based on Span60/Tween80) significantly increased transdermal delivery *in vitro* compared to free drug (Badria and Mazyed, 2020). Similarly, Mehta et al. developed a proniosomal gel (≈708 nm, 98.5% encapsulation efficiency) with 24-h cumulative skin penetration reaching 84.8 mg/cm^2^. It’s *in vivo* anti-inflammatory effect was superior to that of commercial formulations (Mehta et al., 2016). These lipid-based systems improve BA solubilization and surface properties, thus enhancing local absorption and sustained release. Most current studies are limited to *in vitro* and animal models, and further preclinical data are needed.

### 7.6 Cyclodextrin inclusion complexes

Cyclodextrins (CDs) form inclusion complexes with hydrophobic drugs to improve water solubility. Complexes of BA with β-CD or HP-β-CD at molar ratios of 1:1 and 1:2 showed significantly higher release rates in simulated gastric (pH 1.2) and intestinal fluids (pH 6.8) at a 1:2 ratio (Tambe et al., 2018). These results suggest that CD-based carriers can enhance BA dissolution in aqueous media, though *in vivo* pharmacokinetic data are currently lacking.

### 7.7 PEGylation and polymeric carriers

PEGylation of BAs or their covalent linkage to polymers improves solubility and allows for controlled release. A recent report described BA-PEG nanoparticles (∼253 nm) with high stability and spherical morphology. Drug release studies confirmed a sustained-release profile (Thakur and Kiranmai, 2024). In addition, various polymeric carriers (e.g., chitosan, acrylate copolymers, hydrogels) have been explored for BA delivery, offering prolonged release and targeting capabilities. These studies remain preliminary, focusing mainly on physicochemical evaluation and *in vitro* release; further investigation is needed to confirm enhanced bioavailability and efficacy.

#### 7.7.1 Emerging nanotechnologies

Beyond the aforementioned systems, cutting-edge nanotechnology strategies—such as polymeric micelles, metal nanoparticles, metal-organic frameworks (MOFs), and nanogels—have also been applied to BA delivery, reportedly improving oral bioavailability and pharmacodynamic outcomes (Nakhaei et al., 2023). For instance, BAs have been formulated into zinc oxide nanoparticles or incorporated into BA–MOF complexes, both enhancing release and absorption. Collectively, these advanced techniques show great promise at the preclinical level and offer diverse pathways for future application.

### 7.8 Impact of optimization strategies on absorption and anti-inflammatory efficacy

**FIGURE 3:**
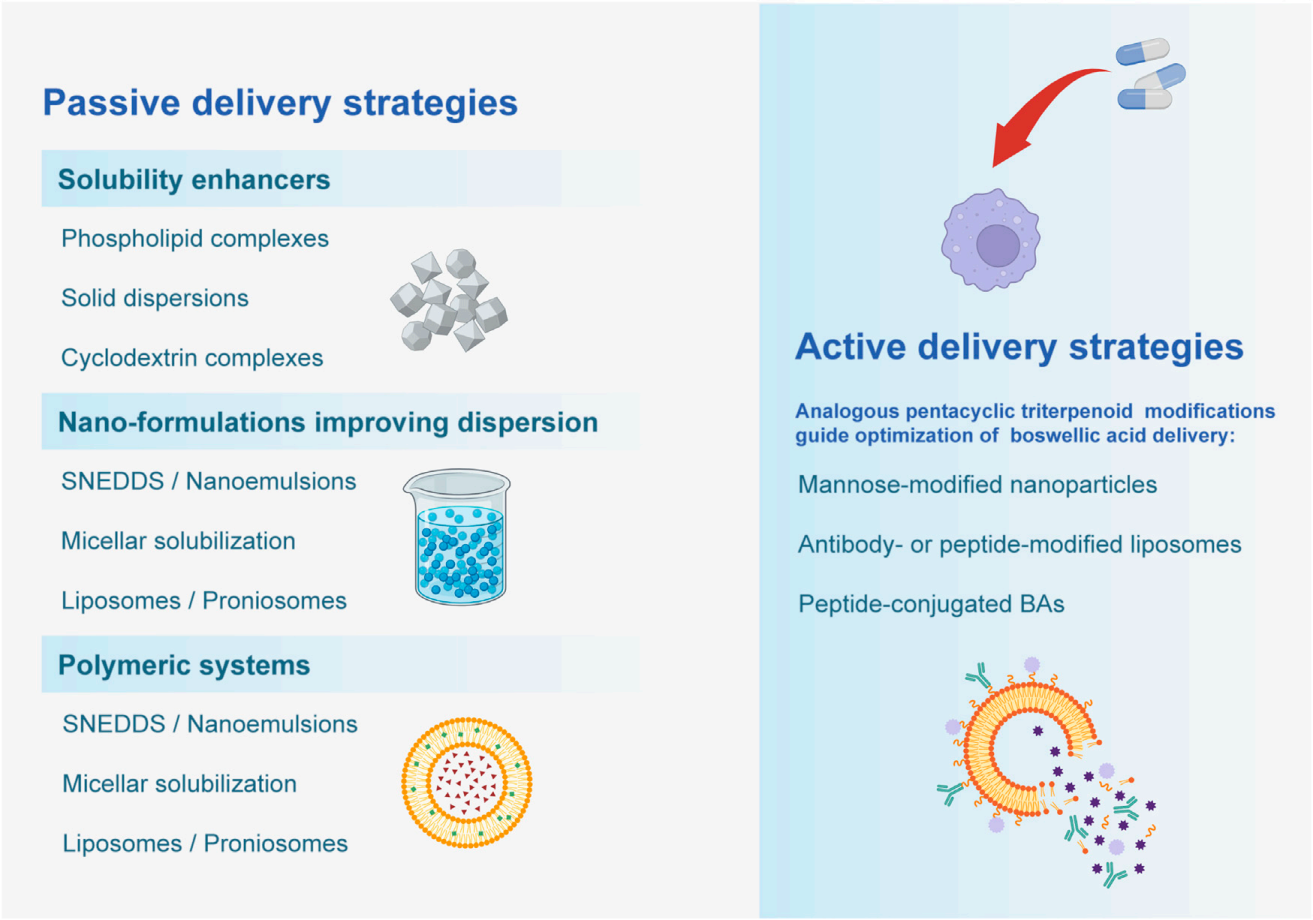
Passive and active delivery strategies for improving Boswellic acid delivery are depicted.

The above strategies have demonstrated significant improvements in both *in vitro* and *in vivo* models. Multiple studies report that optimized formulations significantly enhance water solubility, dissolution rate, membrane permeability, and plasma concentrations of BAs.For instance, solid dispersions and phospholipid complexes have increased BA solubility and *in vitro* release rates by several folds (Tambe et al., 2018; Usapkar et al., 2024). PLGA nanoparticles increased the C_max of AKBA by about 6-fold and AUC by 9-fold (Bairwa and Jachak, 2015). SNEDDS formulations increased KBA/AKBA AUC by approximately 2-fold (Ting et al., 2018); Casperome™ increased KBA AUC by 7-fold (Hüsch et al., 2013). In terms of pharmacodynamics, Nanoformulation generally exhibited stronger therapeutic effects in animal models of inflammation. For example, AKBA-loaded nanoparticles showed significantly greater inhibition in the rat paw edema test compared to free AKBA (Bairwa and Jachak, 2015). BA-Phytosome formulations significantly alleviated joint edema and inflammation in murine arthritis models (Usapkar et al., 2024). These findings demonstrate that enhanced absorption and systemic drug exposure are usually associated with increased anti-inflammatory efficacy (Figure 3).

Although most current formulations of boswellic acids (BAs) focus on passive strategies that improve solubility, dissolution, or absorption, little attention has been paid to active targeting approaches. Active targeting typically involves surface modification of nanocarriers with ligands (e.g., mannose, antibodies, or peptides) to enhance selective uptake by immune cells such as macrophages or inflamed endothelial cells (Lee et al., 2021; Fei et al., 2023; Zheng et al., 2023). Although direct evidence for ligand-targeted nanocarrier delivery of boswellic acids is lacking, analogous nanoparticle systems with other triterpenoids and phytochemicals—such as ursolic acid and asiatic acid loaded into SLNs or nanoparticles—have shown enhanced uptake and anti-inflammatory or neuroprotective effects (Ganesan et al., 2018; Miatmoko et al., 2021; Islamie et al., 2023). These findings support the possibility that ligand-based targeting of BAs may similarly improve their pharmacological performance.” Incorporating such ligand-targeted delivery strategies into BA nanocarrier design could therefore represent a promising future direction to maximize their pharmacological efficacy.

## 8 Discussion

Boswellic acids (BAs), the major active metabolites of frankincense resin, have shown promising therapeutic potential in inflammatory diseases, as demonstrated by numerous *in vitro* and animal studies (Poeckel and Werz, 2006; Ammon, 2010; Ragab et al., 2024). Current research has extensively reported that BAs exert anti-inflammatory, analgesic, antioxidant, and immunomodulatory effects primarily through the inhibition of key signaling pathways and enzymes, such as NF-κB, 5-LOX, COX-2, and iNOS(Werz and Steinhilber, 2005; Abdel-Tawab, 2021; Efferth and Oesch, 2022). In addition, BAs downregulate pro-inflammatory cytokines (e.g., TNF-α, IL-1β, IL-6) and upregulate anti-inflammatory cytokines (e.g., IL-10), exhibiting consistent biological effects across various inflammatory models (Gupta et al., 2001; Ragab et al., 2024).

Despite the substantial progress in preclinical studies, clinical research has not kept pace, particularly in the form of randomized controlled trials (RCTs) utilizing purified BAs such as AKBA and KBA. Most existing clinical studies have employed *Boswellia serrata Roxb. ex Colebr. (Burseraceae)* extract (BSE) as the intervention, which contains a complex mixture of metabolites with undefined BA content (Börner et al., 2021; Majeed et al., 2021). This complexity makes it difficult to attribute therapeutic effects specifically to BAs, thereby limiting the translation of mechanistic findings to human applications. While some RCTs have shown that BSE may provide clinical benefits for conditions such as rheumatoid arthritis, ulcerative colitis, and asthma, these studies are often limited by small sample sizes, variable control settings, short follow-up periods, and a lack of biomarker validation—rendering the evidence insufficient for firm conclusions (Gupta et al., 1997; Gerhardt et al., 2001; Holtmeier et al., 2011; Stürner et al., 2018).

One of the major barriers to the clinical efficacy of BAs is their poor water solubility and low intestinal absorption. Pharmacokinetic studies have demonstrated that both AKBA and KBA exhibit very low oral C_max and AUC values, with plasma levels sometimes undetectable in certain individuals (Hüsch et al., 2013; Kulkarni et al., 2021). Furthermore, their high plasma protein binding significantly reduces the concentration of free drug available for therapeutic action (Krüger et al., 2008). Additional factors such as intestinal efflux mechanisms (e.g., P-glycoprotein) and hepatic metabolism (e.g., CYP3A4) further contribute to their rapid clearance (Cui et al., 2016). These combined challenges make it difficult for orally administered BAs to reach effective systemic concentrations, thereby limiting their pharmacological impact *in vivo*.

To overcome these bioavailability and distribution challenges, a variety of delivery systems have been developed, including Phytosome, PLGA nanoparticles, solid dispersions, self-nanoemulsifying drug delivery systems (SNEDDS), proniosomes, microemulsions/nanoemulsions, and spanlastics for topical use (Sharma et al., 2010; Hüsch et al., 2013; Bairwa and Jachak, 2015; Mehta et al., 2016; Tambe et al., 2018; Ting et al., 2018; Badria and Mazyed, 2020). While these systems have shown marked improvements in pharmacokinetics and therapeutic efficacy *in vitro* and in animal models, most remain at the experimental stage. Issues such as large-scale production, formulation stability, and clinical safety evaluation have yet to be fully addressed. Additionally, there is a lack of comparative studies among these delivery platforms, and indications for specific diseases remain to be clarified (Usapkar et al., 2024).

At present, structural modifications of BAs have been primarily aimed at enhancing their pharmacological activity rather than improving their *in vivo* pharmacokinetic profile. Few studies have investigated chemical modifications designed specifically to optimize the distribution and metabolism of BAs in the human body. This represents a major gap that should be addressed in future research (Csuk et al., 2015; Li et al., 2017; Huang et al., 2018).

It is also important to consider the species-specific differences in BA metabolism. For example, AKBA undergoes deacetylation to KBA in humans, a pathway not observed in rats. In humans, KBA is mainly metabolized by CYP3A4-mediated hydroxylation, while rats possess a distinct enzymatic profile (Krüger et al., 2008; Cui et al., 2016). This discrepancy explains why rodent studies often fail to detect AKBA-derived metabolites, whereas human pharmacokinetic trials report measurable KBA levels following AKBA administration (Reising et al., 2005; Kulkarni et al., 2021).

Such differences indicate that animal models may underestimate the contribution of AKBA metabolism to systemic activity, leading to potential misinterpretation of translational relevance. More importantly, many pharmacological studies still provide insufficient methodological details—such as dose ranges, minimal active concentrations, or the nature of controls—making it difficult to evaluate the validity of reported effects.

Together, these challenges highlight the need for greater reliance on human-relevant models and more rigorous reporting standards to ensure reproducibility and to bridge the gap between preclinical findings and clinical translation.

The Future studies should emphasize the use of humanized liver microsomes, organoids, or integrated gut–liver models to better simulate human metabolic dynamics and improve translational relevance.

In recent years, the gut microbiota has emerged as a major research focus in inflammation and immunity, making it important to consider in the context of boswellic acids (BAs). Although direct evidence remains limited, one study demonstrated that dietary supplementation with Indian frankincense [*Boswellia serrata Roxb. ex Colebr. (Burseraceae)*] resin not only alleviated allergic pulmonary inflammation but also significantly altered gut microbial composition (Suther et al., 2022). These findings suggest that modulation of the gut microbiota could represent an indirect pathway by which BAs exert immunoregulatory effects. Further work integrating microbiome profiling and mechanistic immunology will be essential to clarify this connection.

Moreover, tissue distribution studies have observed accumulation of BAs in the brain and liver (Hüsch et al., 2013). Based on these findings, there has been a growing interest in the application of BAs, particularly AKBA, in central nervous system disorders (Ding et al., 2014; Gong et al., 2022). The ability of BAs to cross the blood–brain barrier and reach effective concentrations in brain tissue has been associated with neuroprotective effects in various neuroinflammation-related models. However, research exploring the hepatic effects of BAs remains limited. Their potential protective roles in liver injury, non-alcoholic steatohepatitis (NASH), and hepatic fibrosis warrant further pharmacological and clinical investigation.

Collectively, current evidence highlights the versatile pharmacological potential of boswellic acids but also underscores persistent challenges related to bioavailability, tissue distribution, and clinical translation. Future investigations should focus on long-term pharmacokinetic profiling, targeted delivery strategies, and large-scale clinical trials to fully elucidate the therapeutic value of these natural triterpenoids.

## Glossary

5-LOX: 5-Lipoxygenase
MAPK: Mitogen-Activated Protein Kinase
IKK: IκB Kinase
iNOS: Inducible Nitric Oxide Synthase
COX-1/2: Cyclooxygenase-1/2
**PGH**_**2**_: Prostaglandin H_2_
Nrf2: Nuclear Factor Erythroid 2–Related Factor 2
ARE(s): Antioxidant Response Element(s)
GPx: Glutathione Peroxidase
SOD: Superoxide Dismutase
CAT: Catalase
HO-1: Heme Oxygenase-1
ECM: Extracellular Matrix
MMP(s): Matrix Metalloproteinase(s)
ROS: Reactive Oxygen Species
RNS: Reactive Nitrogen Species
MDA: Malondialdehyde
PAMPs: Pathogen-Associated Molecular Patterns
DAMPs: Damage-Associated Molecular Patterns
PRRs: Pattern-Recognition Receptors
Bas/BA: Boswellic Acids
AKBA: 3-O-Acetyl-11-keto-β-Boswellic Acid
KBA: 11-keto-β-Boswellic Acid
α-BA/β-BA: Alpha-/Beta-Boswellic Acid
sAKBA: Semi-synthetic Acetoxyl-11-keto-β-Boswellic Acid
EAE: Experimental Autoimmune Encephalomyelitis
OA: Osteoarthritis
IBD: Inflammatory Bowel Disease(s)
RCT: Randomized Controlled Trial
PD: Parkinson’s Disease
PF: Pulmonary Fibrosis
NAFLD: Non-Alcoholic Fatty Liver Disease
NASH: Non-Alcoholic Steatohepatitis
MS: Multiple Sclerosis
MRI: Magnetic Resonance Imaging
CRP: C-Reactive Protein
VAS: Visual Analogue Scale
WOMAC: Western Ontario and McMaster Universities OA Index
LFI: Lequesne Functional Index
CDAI: Crohn’s Disease Activity Index
FEV1: Forced Expiratory Volume in 1 s
FVC: Forced Vital Capacity
PEFR: Peak Expiratory Flow Rate
HLM: Human Liver Microsomes
HIM: Human Intestinal Microsomes
CE2: Carboxylesterase 2
CYP3A4/5/7: Cytochrome P450 3A4/5/7
NMR: Nuclear Magnetic Resonance
MS/MS: Tandem Mass Spectrometry
m/z: Mass-to-Charge Ratio
LC-MS/MS: Liquid Chromatography–Tandem MS
HPLC-ESI/MS: High-Performance LC–Electrospray MS
UHPLC-LTQ-Orbitrap-MS: Ultra-HPLC–LTQ–Orbitrap MS
Cmax: Maximum Plasma Concentration
AUC: Area Under the Curve
NA: Not Analyzed
ND: Not Detectable
SE: Standard Error
SD: Standard Deviation
SNEDDS: Self-Nanoemulsifying Drug Delivery System/Self-Nanoemulsifying System
CDs: Cyclodextrins
β-CD: beta-Cyclodextrin
HP-β-CD: Hydroxypropyl-beta-Cyclodextrin
PLGA: Poly (lactic-co-glycolic acid)
NF-κB: Nuclear Factor kappa-light-chain-enhancer of activated B cells
AP-1: Activator Protein-1
JAK–STAT: Janus Kinase–Signal Transducer and Activator of Transcription
IκB: Inhibitor of κB
ICAM-1: Intercellular Adhesion Molecule-1
VCAM-1: Vascular Cell Adhesion Molecule-1
**PGE**_**2**_: Prostaglandin E_2_
TNF-α: Tumor Necrosis Factor-α
IL-1β: Interleukin-1β
IL-6: Interleukin-6
IL-10: Interleukin-10
IL-17: Interleukin-17
IFN-γ: Interferon-γ
ERK1/2: Extracellular Signal-Regulated Kinase 1/2
TLR: Toll-Like Receptor
IRAK1: Interleukin-1 Receptor-Associated Kinase 1
pIRAK1: phosphorylated IRAK1
Th1/Th2/Th17: T helper 1/2/17
Treg: Regulatory T cell
**CD4** ^**+**^: Cluster of Differentiation 4 positive
GATA3: GATA-Binding Protein 3
FoxP3: Forkhead Box P3
GI: Gastrointestinal
PXM188/407: Poloxamer 188/407
Nat-BE: Native *Boswellia* Extract
Sol-BE: Solubilized *Boswellia* Extract
HLXLD: Huo Luo Xiao Ling Dan
DMM: Destabilization of the Medial Meniscus
ACLT: Anterior Cruciate Ligament Transection
FLS: Fibroblast-Like Synoviocytes
LPS: Lipopolysaccharide
MIA: Mon iodoacetate
DSS: Dextran Sulfate Sodium
HK-2: Human Kidney-2 cell line
LDH: Lactate Dehydrogenase
TGF-β1: Transforming Growth Factor-β1
SMAD2/3/4/7: SMAD family proteins
α-SMA: Alpha-Smooth Muscle Actin
UCP-1: Uncoupling Protein-1
CPT-1: Carnitine Palmitoyltransferase-1
HNE: 4-Hydroxynonenal
LPO: Lipid Peroxidation
NO: Nitric Oxide
GSH: Glutathione
ASA: Acetylsalicylic Acid
HCl: Hydrochloric acid
FCA: Freund’s Complete Adjuvant
NSAIDs: Non-Steroidal Anti-Inflammatory Drugs
GCs: Glucocorticoids
MSM: Methylsulfonylmethane
